# Female Choice Undermines the Emergence of Strong Sexual Isolation between Locally Adapted Populations of Atlantic Mollies (*Poecilia mexicana*)

**DOI:** 10.3390/genes9050232

**Published:** 2018-05-02

**Authors:** Claudia Zimmer, Rüdiger Riesch, Jonas Jourdan, David Bierbach, Lenin Arias-Rodriguez, Martin Plath

**Affiliations:** 1 College of Animal Science & Technology, Northwest A&F University, Yangling 712100, China; mplath-zoology@gmx.de; 2 Department of Ecology and Evolution, Goethe University of Frankfurt, Max-von-Laue-Straße 13, D-60438 Frankfurt am Main, Germany; 3 Centre for Ecology, Evolution and Behaviour, School of Biological Sciences, Royal Holloway University of London, Egham, Surrey TW20 0EX, UK; Rudiger.Riesch@rhul.ac.uk; 4 Department of River Ecology and Conservation, Senckenberg Research Institute and Natural History Museum Frankfurt, D-63571 Gelnhausen, Germany; jonasjourdan@googlemail.com; 5 Department of Biology and Ecology of Fishes, Leibniz-Institute of Freshwater Ecology and Inland Fisheries, Müggelseedamm 310, D-12587 Berlin, Germany; david.bierbach@gmx.de; 6 División Académica de Ciencias Biológicas, Universidad Juárez Autónoma de Tabasco (UJAT), 86150 Villahermosa, Tabasco, Mexico; leninariasrodriguez@hotmail.com; 7 Shaanxi Key Laboratory of Molecular Biology for Agriculture, Northwest A&F University, Yangling 712100, China

**Keywords:** reproductive isolation, ecological speciation, sexual ornament, species discrimination, barrier loci

## Abstract

Divergent selection between ecologically dissimilar habitats promotes local adaptation, which can lead to reproductive isolation (RI). Populations in the *Poecilia mexicana* species complex have independently adapted to toxic hydrogen sulfide and show varying degrees of RI. Here, we examined the variation in the mate choice component of prezygotic RI. Mate choice tests across drainages (with stimulus males from another drainage) suggest that specific features of the males coupled with a general female preference for yellow color patterns explain the observed variation. Analyses of male body coloration identified the intensity of yellow fin coloration as a strong candidate to explain this pattern, and common-garden rearing suggested heritable population differences. Male sexual ornamentation apparently evolved differently across sulfide-adapted populations, for example because of differences in natural counterselection via predation. The ubiquitous preference for yellow color ornaments in poeciliid females likely undermines the emergence of strong RI, as female discrimination in favor of own males becomes weaker when yellow fin coloration in the respective sulfide ecotype increases. Our study illustrates the complexity of the (partly non-parallel) pathways to divergence among replicated ecological gradients. We suggest that future work should identify the genomic loci involved in the pattern reported here, making use of the increasing genomic and transcriptomic datasets available for our study system.

## 1. Introduction

### 1.1. Adaptive Divergence along Ecological Gradients and Reproductive Isolation

A central question in evolutionary biology is how divergent ecological selection—e.g., along ecological gradients [1,2,3]—promotes reproductive isolation (RI) [4,5,6], defining the process of ecological speciation [7,8]. Theory distinguishes between pre- and postzygotic isolating mechanisms, which can act independently or in concert to create varying degrees of RI [5]. However, the genetic basis of these mechanisms is often poorly understood. As a unifying conceptual framework, barrier loci have been suggested, which are either under direct (divergent) selection, part of a functional group of genes that are under pleiotropic selection, or otherwise involved in the development of complex traits that are the target of divergent selection [9]. Considering speciation along ecological gradients, RI can arise when natural selection acts against maladapted individuals migrating between habitat types [10], e.g., because migrants show increased mortality [11,12,13], have lower reproductive fitness [6,8,14], or because hybrid offspring have higher mortality and/or lower reproductive fitness compared to the average offspring of the resident (locally adapted) population [15,16,17,18,19,20].

Another important mechanism of (prezygotic) RI is assortative mating. Here, females (e.g., [21,22]) and/or males (e.g., [23,24,25]) either use specific phenotypic traits characteristic of their own ecotype [26,27,28] or environmentally dependent performance traits [5,29] to choose mating partners from the pool of available mates (for other forms of assortative mating, see [30,31]). While discrimination between species/ecotypes received most attention in the literature on ecological speciation [7,8,31,32], female mate choice actually consists of at least three stages: species identification through species-specific traits and subsequent discrimination in favor of individuals from their own species [21,33,34,35], sex identification and discrimination in favor of males [36,37,38], and quality assessment, once the choosing individual has assessed the former two criteria and identified its counterpart as a potential mate (e.g., [39,40,41,42]). This quality assessment is usually based on variations of quality traits among potential mating partners, including their physical condition [43,44] or secondary sexual characters [45,46]. In some systems, strong preferences for these quality-related traits can override species discrimination, resulting in secondary sexual characters attracting females across species boundaries. For example, females in *Xiphophorus* fishes prefer males of species that show an ornamental caudal “sword” even if their own males lack such a sword [47,48,49,50]. Ancestral female preferences for vertical bar ornamentation [51] and large male body size [33,52,53,54] override species discrimination in other members of the same genus. This can promote hybridization and genomic introgression [55,56]. Therefore, if closely related species or ecotypes live in parapatry, and RI between them is built up to a substantial degree by the aforementioned mechanisms of species/ecotype discrimination, then these systems can be vulnerable to migration of individuals that show such secondary sexual characters.

Male secondary sexual characters—such as male color ornaments used to attract females—are under various forms of ecological selection [57,58,59]. While sexual selection through female mate choice will select for stronger expression of the preferred traits, other selection forces, predominantly natural selection, will shape the traits in other directions. Local adaptation and ecological speciation along certain ecological gradients may shape the expression of male secondary sexual characters, e.g., through differences in nutrient availability rendering these characters important for females to assess male quality in the respective environment. For example, in guppies (*Poecilia reticulata*) and other fishes, male nuptial ornamentation is a reliable signal of male quality, but high predation pressures typically select for reduced male ornamentation [21,57,59,60]. A comparative approach, using replicate population pairs adapted to similar ecological gradients, offers a unique opportunity to investigate whether and how variation in female preferences and male secondary sexual characters affect the emergence of RI [61].

Our present study focuses on just such a system. We examine how habitat-specific evolution of male secondary sexual characters through both natural and sexual selection might either hamper or strengthen the emergence of RI during the early stages of ecological speciation. Specifically, females of the *Poecilia mexicana* species complex exhibit a preference for males from their own ecotypes (based on body shape) over males showing different body shapes [60] across repeated transitions along (and adaptation to) an evolutionarily replicated ecological gradient [62,63]. We demonstrate that a commonly preferred male color ornament appears to bring about a conflict between sexual selection for this particular secondary sexual character and species/ecotype discrimination (see [48,52]).

### 1.2. Ecological Speciation and Reproductive Isolation of H_2_S-Adapted Poecilia spp.

**Figure 1:**
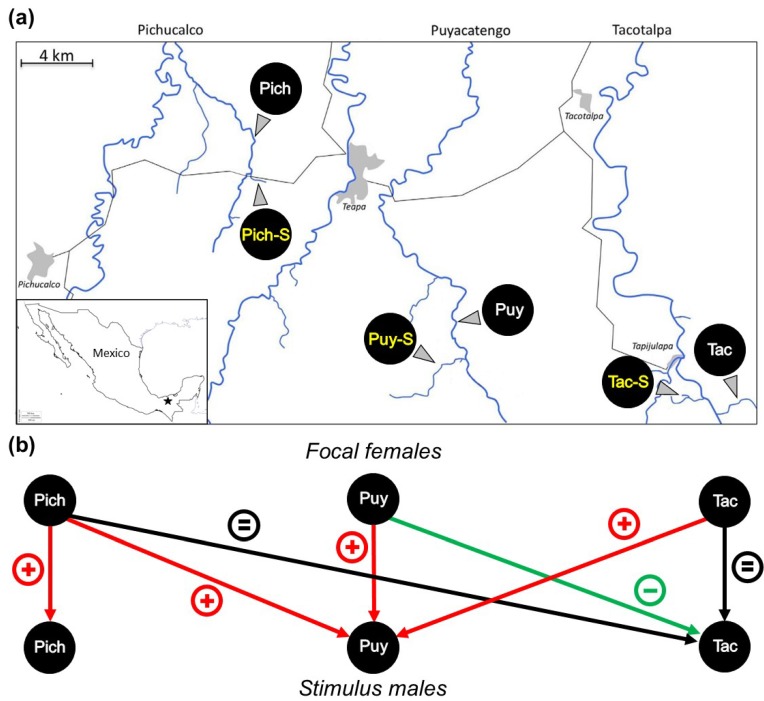
(**a**) Overview of our study area and sampling localities in three parallel rivers in which *Poecilia mexicana* and *Poecilia sulphuraria* were collected (from west to east: Río Pichucalco, Pich = non-sulfidic Río Pichucalco; Pich-S = sulfidic Baños del Azufre; Río Puyacatengo, Puy = non-sulfidic Río Puyacatengo road crossing; Puy-S = sulfidic La Lluvia; Río Tacotalpa, Tac = non-sulfidic Arroyo Bonita, Tac-S = sulfidic El Azufre). All non-sulfidic sites are inhabited by *P. mexicana* populations, while Puy-S and Tac-S harbor locally adapted (sulfide-resistant) *P. mexicana* ecotypes, and Pich-S is inhabited by the closely related, sulfide-endemic *P. sulphuraria*; (**b**) Summary results of binary, dichotomous association preference tests to assess female mating preferences. Focal females from non-sulfidic habitats in all three rivers were given a choice between stimulus males from their own ecotype and the sulfide-adapted ecotype. Results from tests within drainages (vertical arrows) were taken from Plath et al. [12], and we conducted additional cross-drainage tests. The arrows signify a significant deviation of females’ strength of preference (SOP) from zero (two-sided one-sample *t*-tests), suggesting preference for the male phenotype from non-sulfidic habitats (+), no statistically significant preference (=), or preference for the opposite (sulfide-adapted) male phenotype (−). Note that while Puy males were more attractive than Puy-S males within and across rivers, Tac-S males were not less attractive than Tac males, and Puy females even preferred Tac-S over Tac males.

In the southern Mexican states of Tabasco and Chiapas, the neotropical livebearing fish *P. mexicana* has repeatedly colonized toxic, hydrogen sulfide (H_2_S)-containing spring complexes [64]. These sulfidic springs occur in at least four parallel rivers of the Río Grijalva drainage system, and three of these drainages were included in this study (Figure 1a). In the eastern two drainages (Ríos Tacotalpa and Puyacatengo, henceforth Tac and Puy), the H_2_S-rich habitats were colonized relatively recently (<100,000 years ago [65]), and H_2_S-adapted populations (Tac-S and Puy-S) are closely related to—and taxonomically considered the same species as—*P. mexicana* from adjacent non-sulfidic habitats [66]. Sulfide springs in the westernmost drainage (Río Pichucalco, henceforth Pich) have been colonized earlier (>200,000 years ago [65]), and H_2_S-adapted fish (Pich-S) in this drainage were described as a distinct species, the hydrogen sulfide-endemic *Poecilia sulphuraria*, and are more closely related to *Poecilia limantouri*, which is now restricted to northeastern Mexico [66,67,68]. 

Physical barriers to migration are absent, and sulfidic springs in all three rivers directly drain into adjacent freshwater rivers, where H_2_S is immediately diluted [61]. However, migration between different habitat types is strongly impeded. Only sulfide-adapted populations show a tolerance to high concentrations of H_2_S, so that fish from non-sulfidic habitats exhibit high mortality when translocated to sulfidic habitats. Likewise, fish from sulfidic habitats often succumb when transferred to non-sulfidic water [12,62]. All three population pairs in this study show pronounced genetic differentiation, with low levels of gene flow between the ecotypes adapted to sulfidic and non-sulfidic habitats, and RI emerges through both natural (i.e., selection against migrants) and sexual selection, the latter especially driven by females from non-sulfidic habitats discriminating in favor of males from their own ecotype [12,69,70] and by resident males being superior over migrant males in aggressive male contests [71]. 

H_2_S is acutely toxic because of its interference with aerobic respiration [72,73], and populations adapted to high H_2_S concentrations exhibit several shared adaptations to cope with H_2_S and the associated hypoxia [63]. For example, adaptations to H_2_S include morphological changes such as enlarged heads and increased gill surface areas [62,74,75], behavioral adaptations such as increased aquatic surface respiration [76,77,78], and life-history adaptations such as increased offspring size [75,79,80,81]. A comparison of transcriptomic patterns between sulfide-adapted and non-adapted populations revealed differentially expressed genes mainly in the gills, where they affect sulfur and glutathione metabolism, as well as oxidative stress responses [82]. However, a recent focus on the genetic basis of adaptations to H_2_S also revealed genomic [65,68,83,84] and transcriptomic population differences [82,85,86]. For instance, shared evolution of two of the sulfide-adapted populations (Pich-S and Puy-S) was reported for two subunits of the mitochondrially encoded cytochrome-c oxidase complex (COX), which likely offsets the inhibitory function of H_2_S on the mitochondrial respiratory chain [65]. The third H_2_S-adapted population (Tac-S) seems to follow a largely unique evolutionary trajectory, with no sulfide-resistant COX. Moreover, pool-Seq analysis found multiple genome-wide de novo mutations to have been rapidly driven to fixation in Pich-S and Puy-S, but surprisingly little overlap in these mutations was found when comparing both sulfide-adapted populations [84].

As one aspect of sexual selection, mate discrimination has been investigated in the Pich, Puy, and Tac drainages using binary association preference tests [12,69,70]. Mating preferences are likely based, at least to some degree, on the shared morphological changes of sulfide-adapted populations [60,62,74,75]. Still, the results from a previous study [12] investigating mate choice within drainages suggested that (*a*) females, but not males, show strong preferences for mates from their own population over mates from the other ecotype, (*b*) among females, discrimination in favor of males from their own resident population was only seen in populations from non-sulfidic habitats (see also [70]), and (*c*) females’ strength of preference (SOP) showed considerable variation across drainages. The observations (*b*) and (*c*) could partially be explained as an indirect consequence of variation in the strength of natural selection against migrants within and among drainages (estimated using survivorship in 24 h translocation experiments [12,70]). Specifically, assortative mating preferences appear to be stronger when natural selection against migrating males is relatively weak, i.e., when the likelihood of encountering different phenotypes—and, thus, the likelihood of mismatched mating—is high (suggesting reinforcement mechanisms [12,60]). However, some of the variation in the mate choice behavior of females from non-sulfidic waters (effect (*c*) could also arise from the variation in the expression of sexually selected male traits in the different H_2_S-adapted populations. Most strikingly, Tac females did not show a preference for Tac over Tac-S males in the aforementioned study [12]. Tobler et al. [69] used a similar experimental approach, and Tac females again showed no preference for their own ecotype but spent even slightly more time in association with Tac-S males, suggesting that discrimination against the sulfide-adapted ecotype is indeed weak in the Tacotalpa drainage. 

To investigate this further, we asked here if features other than morphological traits render Tac-S males more attractive to *P. mexicana* females from non-sulfidic habitats, thus weakening their preference for their own males. More specifically, the first question we examined in our present study was whether the observed variation in female mate choice for own over sulfide-adapted male phenotypes is caused by differences in females’ intrinsic preferences across river drainages, or whether it can be explained by specific features characteristic of males in the three sulfide-adapted populations (question 1). If the latter was true, then strength of preference (SOP) values in cross-drainage mate choice tests should be a function of the origin of the stimulus males (i.e., which drainage they stem from) rather than a function of the origin of the choosing females. We reanalyzed data on the mate choice behavior of females from non-sulfidic waters [12] and additionally conducted cross-drainage mate choice tests by giving focal females from non-sulfidic habitats of a given drainage a choice between males of both ecotypes from another drainage. A careful interpretation of the summary results (Figure 1b) suggests the following scenario: it is indeed certain features of the Tac-S males that increase their relative attractiveness compared to the Tac males, and it can be inferred that these features likely act in unison with a universal female preference that is shared among populations.

We then compared patterns of male body coloration, because color patterns play a vital role in mate attraction in poeciliid fishes [51,59,87,88,89,90,91,92]. This allowed us to identify the level of shared and unique divergence in male color ornamentation during the repeated transition from non-sulfidic to sulfidic habitats (question 2) [93,94,95]. Using a comparison of color patterns between wild-caught and common-garden reared Tac and Tac-S males, we further asked if the divergence in color patterns between these two populations is caused by phenotypically plastic responses to the H_2_S-toxic habitat or by heritable, evolved, differences (question 3). Finally, we asked which component(s) of this color divergence could explain why males from sulfide-adapted populations are strongly rejected by females from non-sulfidic waters in some, but not all, drainages (question 4).

## 2. Materials and Methods 

### 2.1. Study System and Details on the Study Populations

All populations of *P. mexicana* and *P. sulphuraria* were collected in the vicinity of the city of Teapa in southern Mexico (Figure 1a). Here, the mountains of the Sierra Madre de Chiapas meet the floodplains of northern Tabasco. All three tributaries under investigation eventually drain into the Río Grijalva and are thus interconnected in downstream direction. In the foothills of the Sierra Madre, several sulfide spring complexes are inhabited by *Poecilia* spp. (from west to east: Río Pichucalco (Pich), Río Puyacatengo (Puy), and Río Tacotalpa (Tac)). Hydrogen sulfide in the sulfidic springs in our study area stems from bacterial sulfate reduction in aquifers fed by meteoric water [96]. The sulfur and carbon sources to fuel bacterial metabolism are associated with hydrocarbon deposits [96] and potentially volcanic activity [97]. 

For this study, we captured females only from non-sulfidic sites and males from both habitat types in all three drainages (Figure 1a; Table 1) using a seine (4 m long, 2 mm mesh width) and transported them immediately to our field station in Teapa (i.e., within 60 min upon capture). We kept the fish separated by sex and population in closed and aerated Sterilite containers for up to 3 days prior to the mate choice tests. As we used non-sulfidic water during the mate choice tests, we gradually acclimatized the H_2_S-adapted fish to freshwater conditions by continuously adding small amounts of sulfide-free river water to the containers. Especially in the Tac drainage, high mortality rates of Tac-S fish were observed upon rapid translocation into sulfide-free stream sections in previous studies [12,70], but no mortality was observed in our experiment, probably owing to the gradual change of the water conditions. 

Pictures for the analysis of body coloration were taken from wild-caught males at the site of capture (see below). For the estimation of broad-sense heritability of color differences, we additionally used randomly outbred, mixed-sex laboratory stocks (Tac, Tac-S). These were kept at the animal maintenance facility of Frankfurt University for at least eight generations. Here, the fish lived in large (620 L) population tanks under a 12:12 light/dark cycle and were fed with spinach and chironomid larvae twice a week in addition to daily rations of commercial flake food. This ensured a high availability of dietary carotenoids.

### 2.2. Female Mate Choice within and across Drainages

We reanalyzed the data on the mating preferences of females from non-sulfidic habitats in all three drainages for males from their own ecotype over males from the sulfide-adapted ecotype from Plath et al. [12]. Additionally, we conducted four sets of mate choice tests using stimulus males of both ecotypes (sulfide-adapted and not sulfide-adapted) from a given drainage but used (not sulfide-adapted) focal females from another drainage to find out whether differences in the preferences uncovered during the previously published tests could be explained by intrinsic female traits or could be ascribed to site-specific environmental adaptations of the males (question 1). Binary association preference tests were conducted as described in Plath et al. [12], providing only visual information to the choosing females. This prevented males from approaching females and coercing copulations. In short, focal females were given a choice between two stimulus males (sulfide- and not sulfide-adapted ecotype), and the times spent in association with both males were scored. The test tank (42.6 × 30 × 16.5 cm) was built from UV-transparent Plexiglas and was visually divided into three equally sized zones: the two lateral zones were designated as preference zones, and the central zone as neutral zone. Two smaller auxiliary tanks (19.5 × 30 × 14.5 cm) were placed on either side of the test tank to hold the stimulus fish. The test setup was covered on three sides with light grey cardboard. The observer was sitting quietly in front of the test setup (on the side not covered with cardboard) at a distance of approximately 1.5–2 m. 

To initiate a trial, one stimulus male was placed in each auxiliary tank, and the focal female was introduced into the center of the test tank as soon as the stimulus fish started to swim freely. We granted up to 2 min of acclimatization time, and the measurements started when all fish resumed swimming freely in the water column (they typically froze on the bottom of the test tank for a short period when initially introduced). We measured the time the focal female spent in each preference zone during a 5 min observation period. After the first observation period, the stimuli were switched between sides, and the measurement was repeated to avoid side biases. We altered the side assignments (own or sulfide-adapted ecotype left or right during the first part of the test) for each trial. For each focal female, the association times spent near either stimulus male were summed from both 5 min observation periods, and we measured the standard length (SL) of each fish upon termination of the two test parts. Focal females were tested only once, but a pair of stimulus males was sometimes used in a maximum of two tests, as we encountered problems collecting sufficient numbers of males.

We established strength of preference (SOP) values for each test situation as:

SOP = (time near male from own ecotype − time near sulfide-adapted male)/(time near own male + time near sulfide-adapted male)

SOP values could range from +1 (complete preference for own ecotype) to −1 (complete preference for sulfide-adapted ecotype) and were tested against the ‘no preference’ expectation of SOP = 0 using one-sample *t*-tests. Unless stated otherwise, all statistical tests were run in IBM SPSS Statistics (Version 23.0, IBM Corp., Armonk, NY, USA).

### 2.3. Body Color Divergence in Sulfide-Adapted Populations

Our second question related to population differences in male body coloration. To address this question, we collected adult males between August and September 2011, using seines or dip nets at all six study sites (Table 1; Figure 1a; Appendix A). 

**Figure 2:**
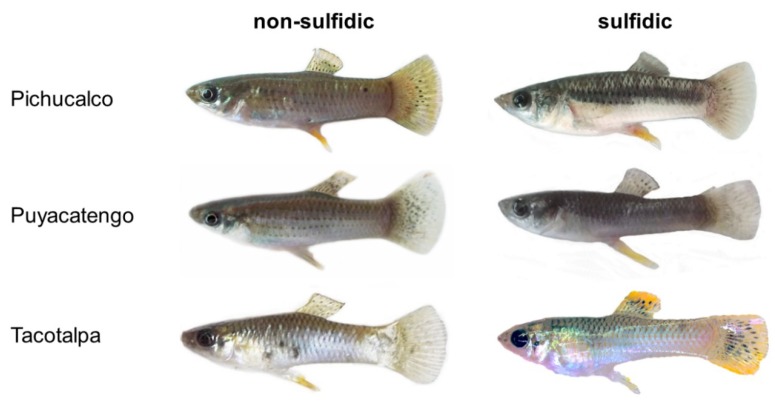
Examples of differences in body coloration of *P. mexicana*/*P. sulphuraria* males from non-sulfidic habitats (left) and the corresponding H_2_S-rich spring in the three focal river drainages in southern Mexico.

**Figure A1:**
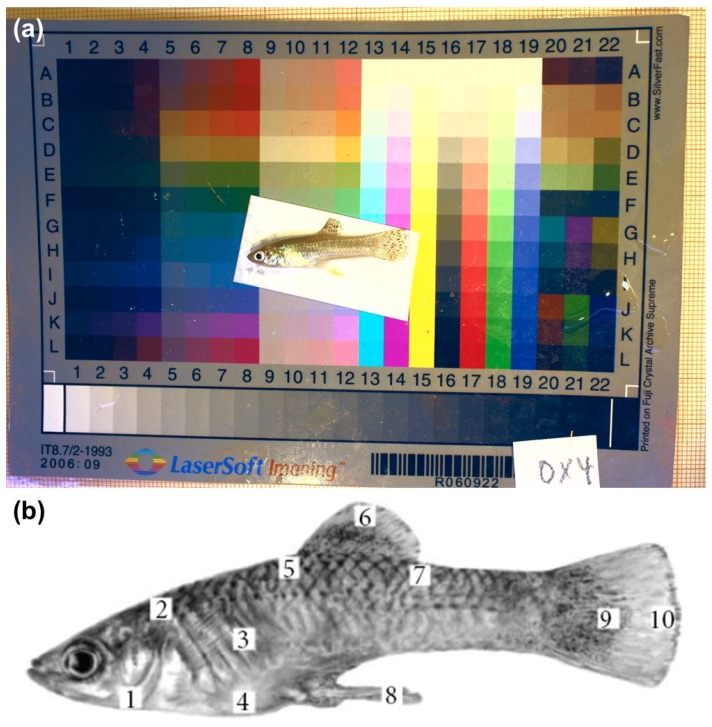
(**a**) Example of a male from a non-sulfidic habitat in the Tacotalpa drainage photographed on the calibration plate; (**b**) illustration of the 10 regions on the body at which color (L*a*b*) measurements were taken.

Unlike in guppies, where males possess distinct, highly variable color spot patterns to attract females [57,59,87,98], coloration in *P. mexicana* is more uniform and based on a beige ground color (Figure 2 and [99]). Large-bodied, dominant males are more conspicuous in body coloration, showing black vertical bars on the body sides along with yellowish to orange color patterns on the margins of the dorsal and caudal fins. Subordinate (mostly smaller-bodied) males are more cryptically colored, with only faint color patterns [99,100]. Although Culumber et al. [101] described occasional black spots in sulfide-adapted fish, we specifically did not include such phenotypes in this study, but attempted to obtain an otherwise representative sample, including both colorful and drab males. We measured body coloration at 10 body regions using Adobe Photoshop (CS5, Adobe Systems Inc., San Jose, CA, USA), following the method described in Bierbach et al. [100]; details can be found in Appendix A, Figure A1. In total, we thus processed 128 images of males (including laboratory-reared males from Tac and Tac-S for the subsequent assessment of broad-sense heritability). We assessed three color metrics (L*a*b* values in CIELAB color space) from all 10 spots, whereby the L* values describe relative lightness ranging from black to white, the a* values describe variations from green to red, and the b* values describe variations from blue to yellow.

To test for differences in body coloration among populations, we condensed the data obtained from wild-caught males by subjecting them to principle component analysis (PCA [100]). Seven PC axes with eigenvalues > 1 (explaining 79.15% of the variance; Appendix A, Table A1 and Table A2) were retained and their PC scores used as dependent variables in a MANCOVA that included drainage and presence of H_2_S as fixed factors and SL as a covariate. We initially included all interaction terms but removed non-significant interactions starting with the highest interaction level in a stepwise backward elimination procedure until a final model was obtained. Post hoc ANCOVAs for each PC score were subsequently conducted (with an otherwise identical structure as the final MANCOVA model) to identify PC scores that contributed to a significant effect in the multivariate model. 

We were specifically interested in shared and unique patterns of body color divergence between male *Poecilia* spp. from sulfidic and non-sulfidic habitats among drainages (question 2). Therefore, we conducted further post hoc analyses for the interaction term of drainage × presence of H_2_S (reflecting drainage-specific divergence in sulfidic habitats). We used residual PC scores from a preparatory MANCOVA (see Appendix A) and conducted multiple comparisons between males from non-sulfidic and sulfidic habitats with independent samples *t*-tests for each drainage and PC score, separately. We corrected the α levels for multiple comparisons using Bonferroni correction. 

### 2.4. Estimating Broad-Sense Heritability of Color Divergence

We sought to disentangle the environmentally induced (i.e., phenotypically plastic) components of color divergence (e.g., as a result of different carotenoid availability in the different sulfide springs, see [102,103,104]), from evolved (i.e., heritable) differences (question 3). To this end, we additionally assessed the body coloration of laboratory-reared *P. mexicana* males from both Tac and Tac-S. We were able to compile a dataset including *N* = 19 laboratory-reared Tac and *N* = 20 laboratory-reared Tac-S males and we included *N* = 12 wild-caught Tac and *N* = 9 wild-caught Tac-S males from our previous analysis (see Section 2.3). A PCA with this data set resulted in six PC axes with eigenvalues > 1 (explaining 80.27% of the variance; Appendix B, Table A4 and Table A5). Axis loadings did not exactly match those of the previous PCA that was based only on wild-caught males (Table A4), but were qualitatively similar. For example, the PC axis containing information about the lightness of the dorsal region was PC 3 in the previous PCA (see above, Section 2.3) but PC 1 in our second PCA; still, both received the highest axis loadings from L* 2, L* 5, and L* 7 (see Appendix A and Appendix B). 

First, we provided an estimate for the degree of (dis)similarity between both populations when comparing wild-caught and laboratory-reared fish by means of cross-validation discriminant function analysis (DFA). We computed prior probabilities while accounting for unequal sample sizes in the dataset. The discriminant functions were calculated using only the data from wild-caught fish (training data set), and the data from the laboratory-reared fish were then inserted into the discriminant functions and assigned to the most parsimonious population of origin (i.e., either Tac or Tac-S). A high classification success would indicate consistent, heritable differences between populations. Additionally, we used intraclass correlation coefficients (ICC, [105]) to pinpoint components of body coloration (in this case PC scores) that show consistent differences across rearing environments, suggesting a broad-sense heritability of those traits (e.g., [106,107]). ICC is based on variance components extracted from an ANOVA and is a measure of repeatability that describes the proportion of variance of a given trait between, rather than within, individuals [108]. ICC values > 0.8 [109] would indicate a high repeatability (i.e., broad-sense heritability) in a trait when comparing lab-reared and wild-caught fish. In other words, both analyses (cross-validation DFA and ICC) have the power to reveal if wild-caught and lab-reared fish within populations exhibited coloration patterns more similar to one another than to fish from the other population.

### 2.5. Does Divergence in Male Coloration Predict Variation in Females’ SOP?

Finally, we asked which component(s) of male body coloration might explain the observed variation in females’ strength of preference (SOP; question 4). We calculated the population differences of the seven PCs derived from the analysis of body coloration in wild-caught males (Section 2.3), i.e., the absolute differences between mean PC scores of the sulfide-adapted ecotype and mean scores of the respective not sulfide-adapted ecotype in a given drainage. We then tested for potential effects of the resulting difference scores on the mean SOP values obtained from the within- and cross-drainage mate choice tests in a multiple linear regression with a stepwise backward elimination procedure. We coded the SOP values as the dependent variables and the difference scores as the predictor variables.

## 3. Results

### 3.1. Female Mate Choice within and among Drainages

**Figure 3:**
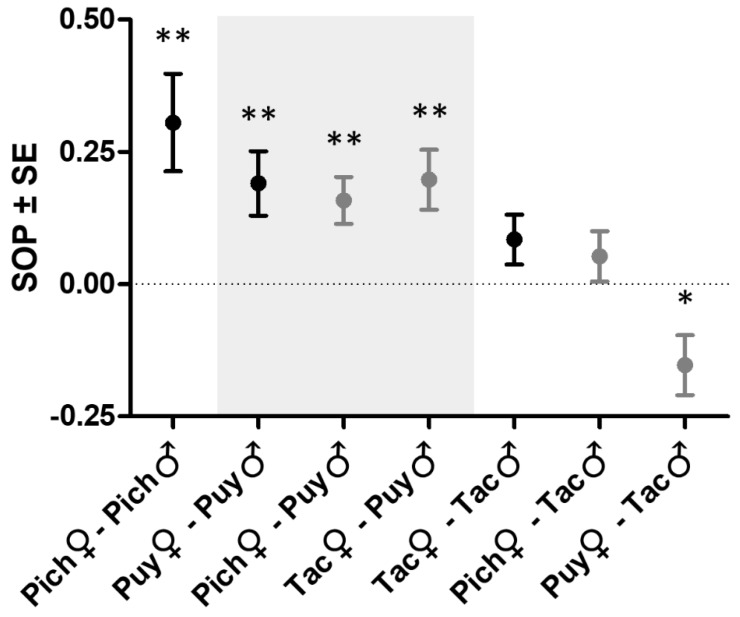
Mean ± SE strength of preference for own over H_2_S-adapted males in *Poecilia* spp. females from non-sulfidic habitats. The tests were conducted within drainages (black symbols; data from Plath et al. [12]) and with stimulus males from another drainage (gray symbols; tests novel to this study). The different backgroud shading groups the tests by the drainage from which the stimulus males were taken. The asterisks indicate significant deviation from the null hypothesis of SOP = 0 (two-sided one-sample *t*-tests; * *p* < 0.05, ** *p* < 0.01).

We presented focal females from non-sulfidic habitats with males of both ecotypes and used the relative association times as a measure of mate discrimination. Pich females showed a strong, statistically significant preference for males from their own ecotype (Figure 3). Likewise, Puy females preferred males from their own ecotype, and very similar (statistically significant) SOP values were found when Pich and Tac females could choose between Puy and Puy-S stimulus males in cross-drainage tests. Presented with males from the Tac drainage, however, the females from all three drainages showed either no preference (in the case of Pich and Tac females) or a statistically significant preference for the sulfide-adapted Tac-S males (Puy females). This resulted in a considerable variation in the SOP values among the seven preference tests, ranging from +0.31 to −0.15. We tentatively argue that the divergence of male characteristics rather than the divergent evolution of female preferences is likely to be the cause of this variation (see Introduction). This rationale formed the basis for our subsequent analyses (Figure 1b). 

### 3.2. Divergence in Male Body Coloration 

MANCOVA, using the seven PC scores from the PCA on male body coloration of wild-caught males from all populations (Appendix A) as the dependent variables, detected significant effects of the covariate SL, the factors drainage and presence of H_2_S, as well as the interaction terms drainage × presence of H_2_S and drainage × SL (Table 2). The strongest effect (based on Wilks’ partial *η*^2^) was seen in the case of the interaction term drainage × presence of H_2_S (reflecting unique, drainage-specific responses to H_2_S-exposure), followed by presence of H_2_S (reflecting shared evolutionary responses to H_2_S-exposure) and the effect of body size (SL; Table 2). Other (weaker) effects are displayed in Appendix A.

**Figure 4:**
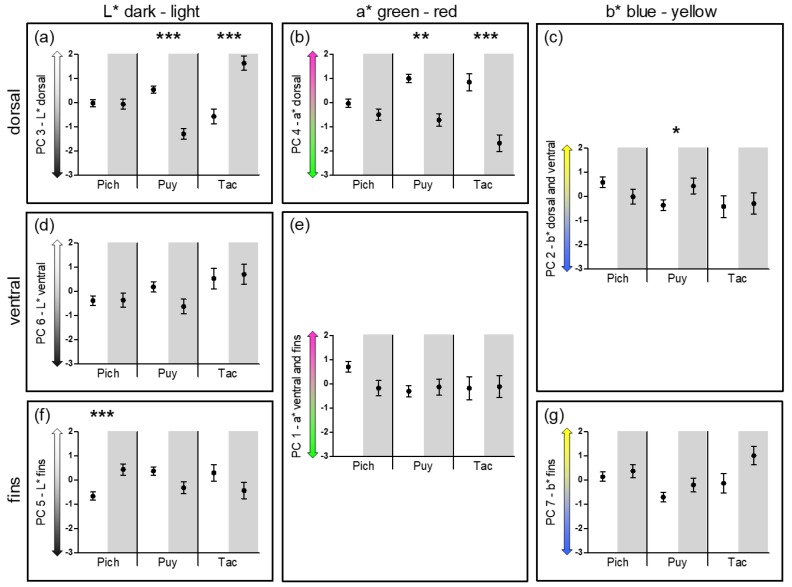
Illustration of the significant effects of drainage × presence of H_2_S detected in post hoc ANCOVAs using principle component (PC) scores as the dependent variables (see Appendix A; Table A3). Shown are residual PC scores ± SE obtained from a preparatory MANCOVA. The results are grouped by body region (dorsal area (**a**–**c**), ventral area (**d**,**e**), and fins (**f**,**g**)) and the color (L*, a*, and b*) captured by the respective PC. In each drainage, white background signifies fish from non-sulfidic habitats, while gray background indicates H_2_S-adapted fish. The asterisks indicate significant results of post hoc independent sample *t*-tests after Bonferroni correction of the α levels (* *p* < 0.017, ** *p* < 0.003, *** *p* < 0.0003).

Post hoc ANCOVAs (Appendix A; Table A3) suggested that the significant interaction effect of drainage × presence of H_2_S was mainly driven by a drainage-specific variation in the lightness of the males’ dorsal region and fin areas (PCs 3 and 5), a variation along the blue-yellow axis on the body (PC 2), and the intensity of red in their dorsal region (PC 4) between sulfide-adapted and non-adapted males. Tac-S males were exceptionally light on their back compared to their counterparts from non-sulfidic waters, while Puy-S males had darker dorsal regions than Puy males (PC 3; Figure 4a). Concerning the lightness of the fins, Pich-S males had lighter fins than males from the corresponding non-sulfidic habitat Pich (PC 5; Figure 4f). Puy-S males showed increased yellow coloration in both dorsal and ventral regions (PC 2; Figure 4c). Moreover, while Tac-S and Puy-S males showed significantly decreased red coloration in their dorsal regions, Pich-S males only showed a trend in this direction, and differences were not statistically significant in this drainage after Bonferroni correction (PC 4; Figure 4b).

Factors contributing to the significant main effect of ‘presence of H_2_S’ were the differences in the intensity of red coloration in the males’ dorsal regions between H_2_S-adapted and non-adapted fish and the lightness of the males’ ventral regions. Fish from sulfidic habitats uniformly showed less red coloration in their dorsal regions (PC 4; Figure 4b) and—mainly driven by differences in the Puy drainage—a darker ventral coloration (PC 6; Figure 4d) than the males from non-sulfidic habitats. 

**Figure 5:**
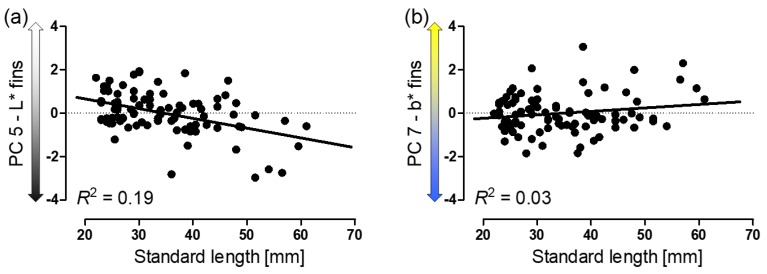
Scatterplots showing the relationships between male body size and (**a**) residual fin lightness (PC 5) and (**b**) residual yellow coloration of the fins (PC 7). Linear regressions and *R*^2^-values are shown to better illustrate the trends.

Body size (SL) significantly affected fin coloration (PCs 5 and 7). The fins of larger males were darker and more yellowish compared to those of smaller males (Figure 5a). However, post hoc Pearson correlations using residuals corrected for presence of H_2_S, drainage, and their interaction term found a significant correlation only in the case of fin lightness (PC 5: *r* = −0.44, *p* < 0.001; Figure 5a; PC 7: *r* = 0.17, *p* = 0.11; Figure 5b). 

### 3.3. Broad-Sense Heritability of Population Differences

We conducted cross-validation DFA using the six body color-related PC scores of our second PCA (Section 2.4). This test only included wild-caught and laboratory-reared Tac males (Appendix B; Table A4 and Table A5) and was conducted to provide a coarse estimate of the broad-sense heritability of ecotype-specific differences in body coloration between Tac and Tac-S males. When attempting to classify laboratory-reared males into their environment of origin (i.e., based on wild-caught Tac and Tac-S males), only 20% of Tac males were classified correctly, but 63.2% of Tac-S males were assigned correctly to their wild-caught counterparts. 

**Figure A4:**
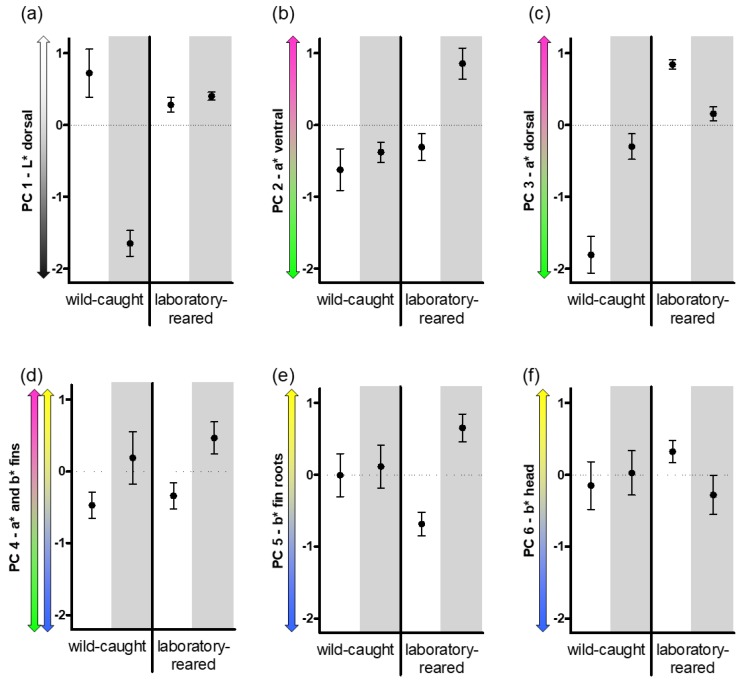
PC scores ± SE used to assess the broad-sense heritability of population differences in male body coloration by comparing wild-caught and laboratory-reared fish from the Tacotalpa drainage. The results are grouped by body region (dorsal area (**a**,**c**), ventral area (**b**), fins (**d**,**e**), and head (**f**)) and the color (L*, a* and b*) captured by the respective PC. The white background signifies fish from non-sulfidic habitats (Tac), while the gray background indicates H_2_S-adapted fish (Tac-S).

The same six PC scores were used to calculate the broad-sense heritability via ICCs. We identified only one PC score with an ICC > 0.8 (Appendix B; Table A6): PC 4, which captures variations in the coloration of the fin tips (a* and b* values of measurement points 6 and 10), showed high consistency between wild-caught and laboratory-reared fish (ICC = 0.991). While laboratory-reared fish of both populations generally had a decreased amount of yellow and red in their fin tips, the difference between H_2_S-adapted and non-adapted fish remained virtually the same (Appendix B; Figure A4d). 

### 3.4. Does Divergence in Male Body Coloration Predict Variation in SOPs?

**Figure 6:**
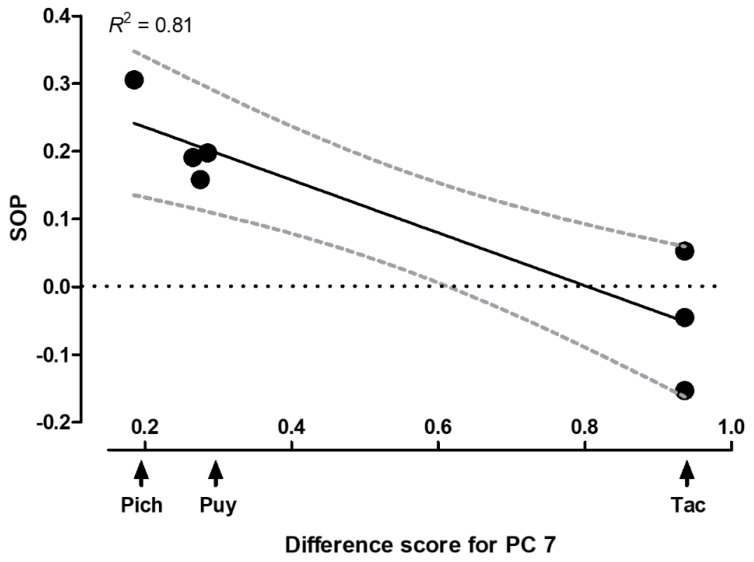
Correlation between females’ SOP for males from their own (not sulfide-adapted) ecotype and the absolute difference between population means in the amount of yellow coloration in males’ fins (PC 7). Shown are data from mate choice tests within and across drainages; for illustration, a linear regression fit and 95% confidence intervals are presented.

In our stepwise backward regression analysis, six PCs had no significant effects and were excluded during the stepwise elimination procedure (PCs 1 and 5: *t* = 1.03, *p* = 0.36; PCs 2‒4: *t* = −1.03, *p* = 0.36). The final model (Nagelkerke *R*^2^ = 0.81) thus only retained PC 7 (*B* = −0.39 ± 0.08, *Beta* = −0.9, *t* = −4.69, *p* = 0.005; Figure 6), which characterizes the intensity of yellow fin coloration. Qualitatively, we obtained the same result when conducting separate linear regression models with only one, or a subset, of predictor variables per model (not shown). A post hoc Spearman rank correlation confirmed a negative relationship between the difference scores for PC 7 and SOP values (*r* = −0.93, *p* = 0.014). Hence, female discrimination against males from the sulfide-adapted ecotype weakened as the intensity of the yellow color ornamentation increased in these males (Figure 6). 

## 4. Discussion

Our present study examined the mate choice component of RI in the *P. mexicana* species complex [12,61,110,111]. Mate choice reduces interbreeding between females from non-sulfidic stream sections and males of the H_2_S-adapted ecotype, which may occur at the interface between sulfidic and non-sulfidic waters [60,64,112]. We focused on mate choice of females from non-sulfidic habitats on the basis that sulfide-adapted males are more likely to briefly venture into adjoining non-sulfidic stream sections, while the opposite migration direction is prevented by migrants not tolerating exposure to high H_2_S concentrations [12,62,70,112]. Furthermore, female mate choice was previously reported as the primary mechanism promoting assortative mating in this system [12,69]. Our cross-drainage preference tests reported here confirmed that females from non-sulfidic habitats generally prefer males from their own ecotype, but that this preference is weakened or reversed in one drainage, namely, the Tacotalpa drainage (question 1). Furthermore, we identified shared and unique differences in coloration between the ecotypes of all three drainages (question 2) and found population differences in one trait, namely, yellow coloration of the fins, to be heritable in Tacotalpa males (question 3). Differences in the same trait could also explain the variation in female preference (question 4). Our present study represents a starting point for future studies that might want to focus on the genetic basis of ecotype differences in male coloration in our study system. 

Mate choice in our study species is mainly based on visual cues [113], but chemical and mechano-sensory cues can also play a role [44], even though chemical communication appears to be more important in certain subterranean (cave-dwelling) populations [114]. Nevertheless, our current experimental design to investigate mate choice preferences offered only visual information to the focal females, and so we may have underestimated the strength of female discrimination in favor of males from their own ecotype. Previous tests demonstrated that the results obtained with experimental setups that allowed full contact between a choosing female and two males (thereby also allowing the assessment of non-visual information) did not differ in the direction of female preferences from those obtained using visual-only setups; at the same time, male behavior had a greater impact on the outcome of the experiment in full-contact scenarios [115]. While mate assessment in our study species is barely ever restricted to visual information in nature, visual courtship displays do not play a major role, and the rate of physical contact between mating partner is very high and, thus, it is classified as sexual harassment [116,117,118]. Females face direct [119] and indirect costs resulting from male harassment [120,121] and tend to avoid direct contact with harassing males [115]. The experimental approach we used here allowed females to choose between two males while avoiding male harassment as well as aggressive interactions between males. 

Considering the results from the within- and cross-drainage mate choice tests, we inferred that the variation in female SOP is based on a visual trait that renders sulfide-adapted males from the Tac-S population relatively more attractive and so we investigated male body coloration (question 2), as color patterns play a central role in the mate choice of poeciliid fishes [59,88,100,122,123,124]. We found both shared and unique (drainage-specific) differences between ecotypes. Besides the morphological traits that H_2_S-adapted males evolved in parallel in different sulfidic spring complexes [60,62,125], any of the shared components of color divergence could be used by the females to discriminate against males of the sulfide-adapted ecotype. Examples include darker ventral regions and the decrease of red coloration on the back of H_2_S-adapted males. In the case of unique patterns of color divergence, the extent of phenotypic divergence between ecotypes differed among drainages, or even opposing trends were uncovered. This high degree of population/drainage-specific divergence mirrors previous studies on other phenotypic traits in this system (e.g., [75]), but has also been reported for various other study systems [126,127]. In our case, any of these uniquely diverging traits could potentially play a role in determining the variation in females’ predilection for own males (e.g., when certain sexually selected traits render sulfide-adapted males relatively more attractive). Future studies could specifically address which components of male body coloration (or combinations thereof) the females actually assess, so as to confirm the interpretations we provide here (e.g., by digital manipulation of color ornaments and subsequent mate choice experiments using computer-animated stimuli [128,129,130,131], but see [132,133,134] for considerations on the applicability of video animations in animal behavior research). An in-depth discussion of all aspects of color divergence and the selective forces driving their divergence lies outside the scope of our present study.

Combining the results from our female mate choice tests and the quantitative results on male body coloration, we made an attempt to identify those male coloration traits that may explain the variation in females’ preference for own males (question 4). Our results suggest that one particular male trait, namely, yellow fin ornamentation, may play a decisive role in determining to what extent the females identify and associate with males from their own ecotype or whether they find the males of the other ecotype relatively more attractive, as those males sometimes display stronger phenotypic trait values (i.e., stronger yellow ornamentation). However, a word of caution is warranted, as the small number of evolutionarily independent replicates (i.e., independently colonized sulfidic spring complexes) in our study system may have led to erroneous interpretations. Future studies could compare the evolution of male color patterns and female mate choice in other poeciliid fishes that have colonized a greater number of sulfidic habitats, such as mosquito fishes of the genus *Gambusia* [75,135]. Moreover, it needs to be considered that we did not detect significant drainage-specific differences between ecotypes in male fin coloration (i.e., the interaction term drainage × presence of H_2_S in post hoc ANCOVA), as would have been expected if drainage-specific differences in sexual selection for this trait drive the observed variation in female preferences. This suggests that additional traits not assessed in this study may also affect the observed variation in female preferences [12,62,70].

Poeciliids, while generally drab and inconspicuous in the female sex [99], have developed various forms of male ornamentation to attract mates and as a signal of dominance [122,136,137]. The females typically prefer brightly colored males, and yellow to orange color ornaments play a central role in determining the outcome of females’ mate assessment [59,88,98,123,138,139]. It has been suggested that male orange color ornaments exploit a pre-existing foraging preference for fruits in guppies [140]. Poeciliids are particularly sensitive to yellow-to-orange colors [130,141]. In fact, the absorption spectra of cones and rods from the retinas of different ecotypes of *P. mexicana* [142] and numerous other poeciliids [143,144,145,146] revealed that they express two visual pigments absorbing at this range of wavelengths. Male orange ornamentation may be influenced by nutrient availability (i.e., algae containing carotenoids [147]) and feeding efficiency [102,124,148] and, thus, can be considered as a condition-dependent trait. In our study, however, we found that of all traits analyzed, differences between ecotypes in male orange fin coloration showed broad-sense heritability (question 3). Specifically, while analyses of body condition [75,81] and general trophic ecology [149,150] suggest that nutrient (and carotenoid) availability is low in H_2_S-rich habitats, population differences in fin coloration persisted after several generations of common-garden rearing with a carotenoid-rich diet (for a similar result in another poeciliid, *Gambusia hubbsi*, see reference [93]). This implies evolutionary divergence, alluding to the existence of (drainage-specific) differences in selective regimes between sulfidic and non-sulfidic waters. Orange displays are not only preferred by females and, thus, under positive sexual selection [59,89,151,152,153], but conspicuous ornamentation also increases predation risk, leading to (natural) counter-selection [57,154]. Sulfidic habitats harbor fewer or no piscine predators [155], but fish in sulfidic habitats face increased areal predation as they engage in aquatic surface respiration [156]. We can only speculate at this point that as yet unknown differences in predator communities and strength of predation among the different sulfidic streams considered here reduce the counter-selection that predation imposes on yellow fin ornamentation especially in the Río Tacotalpa drainage or may affect female preferences for the ornament [157,158,159]. Moreover, drainage-specific effects of the background water color could play an additional role here, which has been reported to affect fin coloration in *G. hubbsi* [93]. Waters rich in oxidized sulfur are generally milky and opaque, and objects in the distance appear blurred, while outlines cannot be discerned easily (similar to fog). Colors seen through this medium loose saturation, and any traits that serve as signals need to be conspicuous [160]. Both dark body coloration and increased intensity of yellow color ornaments could help improve signal transmission/perception in milky water, and the water in the Tacotalpa drainage is indeed particularly milky (see Figure 1 in Plath et al. [161]). We conducted this part of our analysis to shed light on the general evolutionary mechanism (i.e., sexual selection) underlying the observed pattern of variation in male fin ornamentation. We conclude that evolved population differences affect female mate choice for own male phenotypes, but we argue that the heritability of divergence in male color patterns is no prerequisite for a similar pattern to emerge. Habitat-specific differences in carotenoid availability and phenotypic plasticity in the development of male ornaments could lead to a similar pattern in other systems. 

The heritability of population differences in male orange fin ornamentation leads to the question of what the genomic basis of this divergence might be. Future studies into the developmental processes underlying yellow color ornamentation will need to account for the fact that large males in our present study showed darker fins and tended to increase their yellow fin coloration. *P. mexicana* males show a pronounced polymorphism in body size [81,135], and large-bodied males are more likely to establish social dominance [99,162,163]. We propose that both male body size and dominance affect the expression patterns of genes involved in fin coloration, and studies investigating the molecular mechanisms underlying divergence in orange fin ornamentation (e.g., through RNA sequencing) will need to compare males of similar body size. While we forgo speculating about the molecular mechanisms underlying the observed population divergence, future studies looking into the developmental mechanisms involved in this process will certainly benefit from the increasing genomic and transcriptomic toolboxes available for our study system [65,68,82,83,84,85,86]. 

Altogether, we provide evidence that population-specific evolution of male secondary sexual characters can weaken females’ tendency to discriminate in favor of males from their own ecotype, which may hamper the emergence of strong(er) RI. Nevertheless, previous studies found the rates of gene flow to be low, particularly in the Tac drainage [12], indicating that additional mechanisms prevent extensive introgression and hybridization. Indeed, not only direct selection against maladapted individuals affects migrant fitness [12,70], but also predation, for instance, by giant water bugs [164,165,166]. Likewise, sexual selection is made up not only by female choice but also by migrant males being inferior in achieving social dominance during aggressive encounters with resident males [71]. Our study is of general interest, as variable degrees of RI are regularly reported even during repeated transitions and adaptations along similar ecological gradients [167,168,169]. Parts of this variability can be explained by, for example, mutation-order effects or fluctuation in selective regimes (reviewed by [169]), or by gradual variation in the strength of divergent selection [12,75]. However, the possibility remains that sexual selection processes weakening female preferences for own (locally adapted) male phenotypes also contribute to the observed variability in the strength of RI [170,171,172,173], and we argue that mechanisms as exemplified in our present study could actually be widespread and could also apply to other model systems of ecological speciation (see also [173]). 

## Figures and Tables

**Table 1:** Study sites, sample sizes (*N*), and standard lengths (SL) of *Poecilia* spp. collected for the analyses of male body coloration and cross-drainage mate choice tests.

Drainage	Site (Abbreviation)	Male Body Coloration (*N*)	Mean SL ± SD	Mate Choice Tests (*N*, Females/Males)	Mean SL ± SD (Females/Males)
Pichucalco	Baños del Azufre (Pich-S)	12	28.3 ± 3.7	-/30	-/27.3 ± 3.2
	Río Pichucalco (Pich)	19	35.8 ± 9.6	73/30	41.4 ± 12.5/28.6 ± 3.8
Puyacatengo	La Lluvia (Puy-S)	13	24.9 ± 1.8	-/59	-/29.8 ± 3.9
	Río Puyacatengo (Puy)	24	40.6 ± 9.8	52/59	38.3 ± 10.7/33.1 ± 3.7
Tacotalpa	El Azufre (Tac-S)	9	27.6 ± 2.2	-/56	-/34.3 ± 5.4
	Arroyo Bonita (Tac)	12	42.8 ± 6.1	51/56	38 ± 8.4/33.1 ± 5.4

**Table 2:** Results from MANCOVA using principal components (PCs) based on L*a*b* values of 10 spots on the body of male *Poecilia* spp. as dependent variables. *F*-ratios and effect strengths (partial *η*^2^) were approximated using Wilk’s *λ*. SL: standard length.

Effect	*F*	Hypothesis *df*, Error *df*	*p*	Wilks’ Partial *η*^2^
SL	6.876	7, 74	<0.001	0.394
drainage	2.384	14, 148	0.005	0.184
presence of H_2_S	7.663	7, 74	<0.001	0.420
drainage × SL	2.359	14, 148	0.006	0.182
drainage × presence of H_2_S	8.632	14, 148	<0.001	0.450

**Figure A2:**
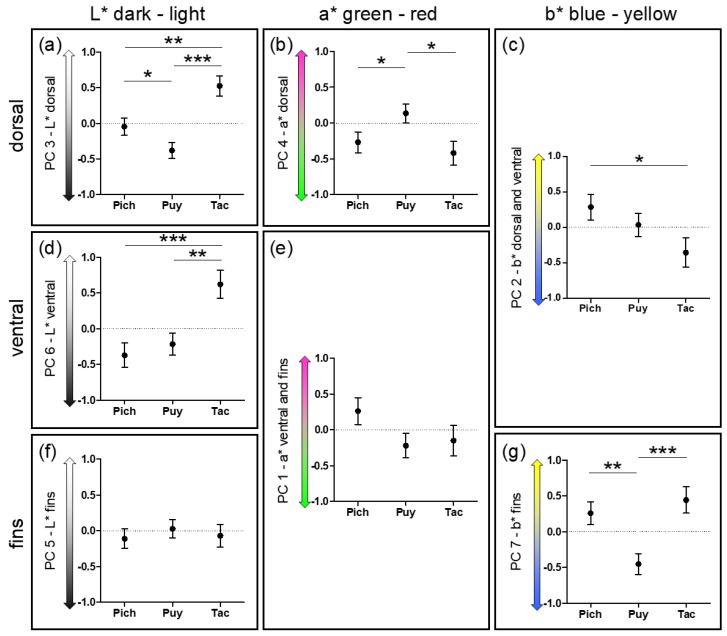
Illustration of the significant effect of ‘drainage’ detected in the main MANCOVA model using PC scores as dependent variables (see Table 2). Shown are estimated marginal means ± SE obtained from post hoc ANCOVAs. The results are grouped by body region (dorsal area (**a**–**c**), ventral area (**d**,**e**), and fins (**f**,**g**)) and the color (L*, a* and b*) captured by the respective PC. The asterisks indicate significant results of the post hoc pairwise comparisons (Fisher’s LSD; * *p* < 0.05, ** *p* < 0.01, *** *p* < 0.001).

**Figure A3:**
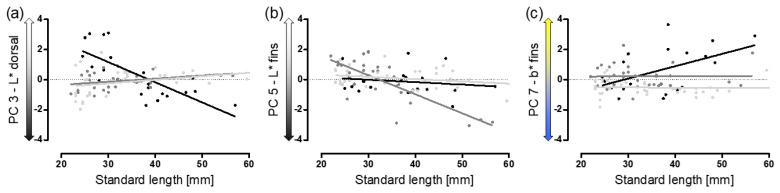
PC scores that showed a significant drainage × SL effect in ANCOVAs. Shown are values for Tac (black), Pich (dark gray), Puy (light gray) for (**a**) PC 3; (**b**) PC 5; and (**c**) PC 7.

## Appendix A

### Appendix A.1. Photographs and Color Adjustment of Pictures

Immediately after capture, fish were anesthetized using MS222 to ensure that melanophores would be relaxed, i.e., maximally opened, and full coloration was measured. Fish were laid on a laminated piece of white paper, which was placed on a laminated color calibration plate (IT8.7/2 LaserSoft Imaging, ID No. R051025; Figure A1a), and photographed from centrally above at approximately 30 cm distance using a Fujifilm Finepix AX250 14MP digital camera. All photographs were taken while avoiding direct sunlight to minimize reflections. We processed the images in random order within and across sites. Prior to the color measurements, we used the gradation curve modulation in Adobe Photoshop CS5 and calibrated each image according to the manufacturer’s instructions (LaserSoft Imaging, Kiel, Germany). The photographs were initially saved as .jpeg files (with RGB color space) but converted to *L*a*b** (CIELAB) color space and saved as .psd files using Adobe Photoshop (CS5, Adobe Systems Inc., San Jose, CA, USA). We adjusted the black-and-white squares as well as squares L13 to L19 of the calibration plate to the provided (standardized) *L*a*b** values. This procedure ensured that each photograph had the same standard coloration and thus allowed for a quantitative comparison of individual color differences.

Each square in which body coloration was quantified measured 1/35 of the fish’s standard length (SL), and measurements represent the average values for each square of the three coordinates in the *L***a***b** (CIELAB) color space, i.e., the lightness of the image (where *L** = 0 represents black and *L** = 100 represents white), its position between green (*a** = −150) and red/magenta (*a** = +100), and its position between blue (*b** = −100) and yellow (*b** = +150). The ten squares were chosen to cover different body regions, with three squares being situated dorsally, three on the ventral side, three on the fins, and one laterally beneath the pectoral fin (Figure A1b). For each photographed fish, SL and body height (maximum distance from dorsal to ventral sides) were also taken using the ruler function of Adobe Photoshop, while taking a reference measurement from millimeter paper visible on the image (Figure A1a).

### Appendix A.2. Principal Component Analysis to Analyze the Divergence of Male Body Coloration

All analyses were conducted using IBM^©^ SPSS^©^ 23. Prior to the analyses, the data were checked to meet the requirements of normal distribution and homoscedasticity using Shapiro-Wilks’ and Levene’s tests, respectively.

The PCA to investigate divergence of male body coloration using wild-caught males from all populations considered in this study resulted in seven PC axes with eigenvalues > 1 (Table A1); axis loadings were Varimax-rotated (see Table A2 for loadings). For better interpretation, axis loadings > 0.75 of the different measurement points were used in ‘identifiers’ to indicate the body regions [‘dorsal’ (2, 5, 7), ‘ventral’ (1, 4), and ‘fins’ (6, 9, 10); points 3 and 8 had no strong correlation to either region] as well as the color components contained in each PC score (Table A1).

**Table A1:** PC scores with eigenvalues > 1 retained from a PCA on *L***a***b** values of ten spots on the body surface of male *Poecilia* spp. collected in sulfidic and non-sulfidic waters. For better interpretation of PC scores, the body regions (dorsal, ventral, fins) and their corresponding *L***a***b** values contained in a given PC are summarized (‘identifier’) on the basis of axis loadings > 0.75 of the respective PC (the numbers refer to the ten areas on the body for which coloration was quantified).

PC	Eigenvalue	Percent Variance Explained	Loadings > 0.75	Identifier
1	6.573	21.911	*a** 3, 4, 8, 9	*a** ventral and fins
2	5.420	18.066	*b** 2, 3, 5, 7	*b** dorsal and ventral
3	3.640	12.134	*L** 2, 5, 7	*L** dorsal
4	2.948	9.826	*a** 2, 5, 7	*a** dorsal
5	2.174	7.248	*L** 10	*L** fins
6	1.703	5.675	*L** 1,4	*L** ventral
7	1.285	4.285	*b** 6, 10	*b** fins

**Table A2:** Axis loadings of the principle component analysis (PCA) analyzing male body coloration (wild-caught males from all populations considered in this study) after Varimax rotation. Axis loadings > 0.75 are highlighted in boldface.

Variable	PC 1	PC 2	PC 3	PC 4	PC 5	PC 6	PC 7
*L** 1	−0.04	−0.20	0.01	−0.14	<0.01	0.87	0.07
*a** 1	0.66	−0.01	0.16	0.26	0.06	−0.43	0.23
*b** 1	0.10	0.57	−0.02	0.05	0.31	−0.44	0.30
*L** 2	0.08	0.11	**0.90**	−0.25	0.08	0.04	−0.05
*a** 2	0.35	<0.01	−0.30	**0.80**	−0.13	−0.05	−0.06
*b** 2	0.04	**0.81**	0.27	−0.17	−0.07	−0.20	0.11
*L** 3	−0.27	−0.06	0.47	0.55	0.09	0.10	−0.10
*a** 3	**0.86**	0.08	0.02	0.14	−0.09	−0.01	0.02
*b** 3	0.12	**0.85**	0.03	0.06	−0.14	−0.03	0.02
*L** 4	−0.15	−0.18	0.28	0.22	0.16	**0.80**	0.02
*a** 4	**0.82**	0.07	−0.05	0.21	−0.11	−0.22	0.21
*b** 4	0.09	0.58	−0.32	0.03	0.17	−0.02	0.50
*L** 5	0.02	0.11	**0.93**	−0.08	0.09	0.08	−0.05
*a** 5	0.47	<0.01	−0.21	**0.82**	0.02	−0.04	−0.05
*b** 5	0.01	**0.87**	0.23	0.03	0.03	−0.24	−0.11
*L** 6	−0.06	−0.10	0.33	−0.07	0.64	<0.01	0.10
*a** 6	0.59	−0.36	<0.01	0.31	−0.30	0.09	0.32
*b** 6	−0.05	0.05	−0.03	−0.14	0.09	0.05	**0.89**
*L** 7	−0.04	0.17	**0.88**	−0.09	0.27	<0.01	−0.04
*a** 7	0.52	<0.01	−0.19	**0.76**	0.06	0.07	−0.08
*b** 7	−0.06	**0.86**	0.20	0.06	0.13	−0.12	−0.13
*L** 8	−0.28	−0.17	−0.01	0.34	0.47	0.54	−0.01
*a** 8	**0.83**	0.21	−0.02	0.01	<0.01	<0.01	−0.05
*b** 8	0.13	0.45	−0.32	−0.16	0.41	0.38	−0.16
*L** 9	−0.03	0.14	0.40	0.34	0.66	0.19	−0.24
*a** 9	**0.89**	0.05	−0.04	0.07	−0.13	−0.02	−0.10
*b** 9	0.25	0.59	−0.25	−0.19	0.27	0.19	0.03
*L** 10	−0.16	0.13	0.07	−0.02	**0.77**	0.05	−0.05
*a** 10	0.43	−0.35	0.01	0.31	−0.51	−0.02	0.41
*b** 10	0.14	−0.03	−0.06	−0.04	−0.28	−0.04	**0.89**

### Appendix A.3. Additional Results on Population Differences in Male Body Coloration (Wild-Caught Males)

Following the MANCOVA analysis (Table 2), we conducted separate post hoc ANCOVAs to explore the contribution of each PC score to the significant effects in the main model (Table A3). Similar to the MANCOVA, the initial models included all interaction terms that were removed step-wise if non-significant, starting with the highest-level interactions.

**Table A3:** Post hoc ANCOVAs for each PC separately. *F*-ratios and effect strengths (partial *η*^2^) were approximated using Wilk’s *λ*. The statistically significant effects are highlighted in bold.

Effect	*F*	Hypothesis *df*, Error *df*	*p*	Wilks’ Partial *η*^2^
PC 1 (*a** ventral, *a** fins)				
**drainage**	**3.992**	**2, 85**	**0.022**	**0.086**
presence of H_2_S	2.051	1, 85	0.156	0.024
PC 2 (*b** body)				
**drainage**	**3.578**	**2, 83**	**0.032**	**0.079**
presence of H_2_S	2.566	1, 83	0.113	0.030
**drainage × presence of H_2_S**	**3.991**	**2, 83**	**0.022**	**0.088**
PC 3 (*L** dorsal)				
**drainage**	**3.307**	**2, 80**	**0.042**	**0.076**
presence of H_2_S	0.232	1, 80	0.632	0.003
SL	1.363	1, 80	0.246	0.017
**drainage × presence of H_2_S**	**24.432**	**2, 80**	**<0.0001**	**0.379**
**drainage × SL**	**3.645**	**2, 80**	**0.031**	**0.084**
PC 4 (*a** dorsal)				
**drainage**	**5.666**	**2, 83**	**0.005**	**0.120**
**presence of H_2_S**	**47.342**	**1, 83**	**<0.0001**	**0.363**
**drainage × presence of H_2_S**	**4.486**	**2, 83**	**0.014**	**0.098**
PC 5 (*L** fins)				
**drainage**	**3.663**	**2, 80**	**0.030**	**0.084**
presence of H_2_S	0.182	1, 80	0.671	0.002
**SL**	**20.969**	**1, 80**	**<0.0001**	**0.208**
**drainage × presence of H_2_S**	**9.441**	**2, 80**	**<0.0001**	**0.191**
**drainage × SL**	**3.864**	**2, 80**	**0.025**	**0.088**
PC 6 (*L** ventral)				
**drainage**	**9.380**	**2, 85**	**<0.0001**	**0.181**
**presence of H_2_S**	**6.826**	**1, 85**	**0.011**	**0.074**
PC 7 (*b** fins)				
**drainage**	**4.630**	**2, 82**	**0.012**	**0.101**
presence of H_2_S	3.367	1, 82	0.070	0.039
**SL**	**10.811**	**1, 82**	**0.001**	**0.116**
**drainage × SL**	**7.575**	**2, 82**	**0.001**	**0.156**

We also found significant effects of drainage and drainage × SL in our main model (i.e., MANCOVA; Table 2a). We calculated estimated marginal means (EMMs) from post hoc ANCOVAs on single PCs on the basis of the model structure of the main MANCOVA for a hypothetical average-sized male with a standard length of 34.6 mm and performed pairwise comparisons for each PC score to investigate the effects of drainage. Even though ANCOVAs showed significant differences between drainages for all PC scores (Table A3), pairwise comparisons with EMMs did not find significant differences in the lightness of the fins (PC 5; Figure A2f) or the red coloration of the fins and the abdomen (PC 1; Figure A2e). By contrast, all coloration aspects of the dorsal region (PC 2, PC 3, and PC 4), the lightness of the ventral region (PC 6), and the yellow coloration of the fins showed differences among drainages. Compared to Pich males, Tac males showed less yellow body coloration (PC 2; Figure A2c) and lighter abdominal regions; the latter was also true in comparison to Puy males (PC 6; Figure A2d). All three drainages differed in dorsal lightness, with Tac males having the lightest, and Pich males the darkest dorsal regions (PC 3; Figure A2a). Puy males showed significantly more red in their dorsal coloration (PC 4; Figure A2b) and less yellow in their fins (PC 7; Figure A2g) than males from the other two drainages.

To further examine the interaction effect of ‘drainage × SL’, we used the original PC scores as dependent variables and drainage as a fixed factor in ANCOVA models to extract unstandardized residuals. We calculated Pearson correlations between residual PC scores and SL for each drainage. In Tac males, we detected a significant correlation between SL and dorsal lightness (PC 3; Figure A3a), with larger males having darker dorsal regions (Tac: *r*_p_ = 0.56, *p* < 0.0001; Pich and Puy: *r*_p_ < 0.1, *p* > 0.06), and between SL and yellow fin coloration (PC 7; Figure A3c), with yellow coloration increasing with body size in Tac (*r*_p_ = 0.26, *p* = 0.018) but not in Pich and Puy (*r*_p_ < 0.01, *p* > 0.8). Regarding the lightness of the fins (PC 5; Figure A3b), larger Pich males showed darker fins (Pich: *r*_p_ = 0.59, *p* < 0.0001; Tac and Puy: *r*_p_ < 0.07, *p* > 0.1).

## Appendix B

### Broad-Sense Heritability

To investigate the heritability of the color patterns in the Tac drainage, we conducted another PCA with wild-caught and laboratory-reared males. The PCA resulted in six PC axes with eigenvalues > 1 (Table A4). Again, axis loadings were Varimax-rotated (see Table A5 for details of axis loadings). Similar to the previous PCA that included wild-caught fish from all populations investigated in this study, we combined information from axis loadings into ‘identifiers’ to allow for a more intuitive interpretation of the involved body region(s) and color component(s) [‘dorsal’ (2, 5, 7), ‘ventral’ (1, 4), and fins (6, 9, 10); points 3 and 8 had no strong correlation to either region; Table A4]. PC scores are visualized in Figure A4. We used population means to calculate ICCs in separate models (with identical model structures) for each PC score (Table A6).

**Table A4:** PC scores with eigenvalues > 1 that were retained from a preparatory PCA and were used to assess the broad-sense heritability of population differences in male body coloration in the Tacotalpa drainage. For better interpretation of PC scores, the body regions and their corresponding *L***a***b** values contained in a given PC are summarized (identifier) based on axis loadings > 0.75 of the respective PC.

PC	Eigenvalue	Percent Variance Explained	Loadings > 0.75	Identifier
1	8.966	29.888	*L** 2, 5, 7	*L** dorsal
2	4.892	16.307	*a** 3, 9	*a** ventral
3	3.867	12.889	*a** 2, 5, 7, *L** 8	*a** dorsal
4	2.760	9.201	*a** 10, *b** 6, 10	*a** and *b** fins
5	2.325	7.751	*b** 8, 9	*b** fin roots
6	1.271	4.238	*b** 1	*b** head

**Table A5:** Axis loadings of the preparatory PCA used to assess the broad-sense heritability of male body coloration after Varimax rotation. Loadings > 0.75 are highlighted in boldface.

Variable	PC 1	PC 2	PC 3	PC 4	PC 5	PC 6
*L** 1	−0.63	−0.40	−0.37	−0.06	0.19	−0.02
*a** 1	0.37	0.72	0.30	0.18	−0.15	−0.03
*b** 1	0.29	0.08	0.12	0.19	0.10	**0.81**
*L** 2	**0.90**	0.09	−0.28	−0.07	0.08	0.06
*a** 2	−0.22	0.23	**0.77**	0.09	−0.11	0.33
*b** 2	0.57	0.22	−0.07	0.18	0.57	0.36
*L** 3	0.51	0.20	0.72	0.11	−0.06	−0.02
*a** 3	0.10	**0.85**	0.10	0.17	0.16	−0.04
*b** 3	0.23	−0.36	0.34	−0.10	0.11	0.61
*L** 4	−0.18	−0.71	0.19	−0.28	−0.26	0.06
*a** 4	−0.21	0.72	0.06	0.09	0.12	−0.04
*b** 4	−0.06	0.12	0.17	0.32	0.36	0.69
*L** 5	**0.94**	0.07	−0.06	−0.06	0.02	0.13
*a** 5	−0.11	0.35	**0.78**	0.20	−0.08	0.36
*b** 5	0.64	0.22	0.15	0.17	0.48	0.42
*L** 6	0.60	−0.33	−0.05	0.44	0.15	−0.03
*a** 6	0.04	0.55	0.30	0.61	−0.15	0.06
*b** 6	0.10	0.19	−0.01	**0.87**	0.30	−0.01
*L** 7	**0.95**	0.08	0.09	−0.06	<0.01	<0.01
*a** 7	−0.07	0.43	**0.76**	0.15	−0.05	0.32
*b** 7	0.62	0.23	0.31	0.12	0.49	0.34
*L** 8	−0.01	−0.27	**0.83**	0.04	0.20	−0.15
*a** 8	0.07	0.71	−0.03	−0.16	0.12	0.36
*b** 8	−0.13	0.14	−0.10	−0.21	**0.88**	0.14
*L** 9	0.57	−0.09	0.56	−0.27	0.11	0.07
*a** 9	0.25	**0.77**	0.17	0.25	0.26	0.10
*b** 9	0.15	0.18	<0.01	0.09	**0.89**	0.09
*L** 10	0.36	−0.25	0.42	−0.16	−0.20	0.15
*a** 10	−0.13	0.32	0.23	**0.78**	−0.22	0.11
*b** 10	−0.06	0.07	−0.02	**0.86**	−0.05	0.25

**Table A6:** Intraclass correlation coefficients (ICC) calculated to provide estimates of the broad-sense heritability of population differences in male body coloration in the Tacotalpa drainage. We calculated population means for each PC score and ran a 2-way mixed effects model, testing for consistency [105]. The values indicating high consistency (ICC > 0.8 [109]) are highlighted in boldface.

PC	ICC	*F* (*df* = 1)	*p*
1	−0.225	0.816	0.532
2	0.575	2.352	0.368
3	−6.089	0.141	0.771
**4**	**0.991**	**105.307**	**0.062**
5	0.305	1.440	0.442
6	−2.331	0.300	0.681
